# Unravelling the crystal structure of Nd_5.8_WO_12−δ_ and Nd_5.7_W_0.75_Mo_0.25_O_12−δ_ mixed ionic electronic conductors

**DOI:** 10.1107/S1600576720012698

**Published:** 2020-10-26

**Authors:** Tobias Scherb, Andrea Fantin, Stefano Checchia, Christiane Stephan-Scherb, Sonia Escolástico, Alexandra Franz, Janka Seeger, Wilhelm A. Meulenberg, Francesco d’Acapito, José M. Serra

**Affiliations:** a Helmholtz-Zentrum-Berlin für Materialien und Energie GmbH, Hahn-Meitner-Platz 1, Berlin 14109, Germany; b Technische Universität Berlin, Hardenbergstrasse 36, Berlin 10623, Germany; c European Synchrotron Radiation Facility (ESRF), 71 avenue des Martyrs, Grenoble 38043, France; d Bundesanstalt für Materialforschung und -prüfung, Unter den Eichen 87, Berlin 12205, Germany; e Freie Universität Berlin, Malteserstrasse 74-100, Berlin 12249, Germany; f Instituto de Tecnología Química (Universitat Politècnica de València-Consejo Superior de Investigaciones Cientifícas), Avenida Los Naranjos s/n, Valencia 46022, Spain; g Forschungszentrum Jülich GmbH, Jülich 52425, Germany; h CNR-IOM-OGG c/o ESRF, LISA CRG, 71 avenue des Martyrs, Grenoble 38043, France

**Keywords:** powder diffraction, mixed conductors, X-ray absorption spectroscopy (XAS), Nd_6−*y*_WO_12−δ_

## Abstract

Mixed ionic electronic conducting ceramics Nd_6−*y*_WO_12−δ_ (δ is the oxygen deficiency) provide excellent stability in harsh environments containing strongly reactive gases such as CO_2_, CO, H_2_, H_2_O or H_2_S. Due to this chemical stability, they are promising and cost-efficient candidate materials for gas separation, catalytic membrane reactors and protonic ceramic fuel cell technologies. As in La_6−*y*_WO_12−δ_, the ionic/electronic transport mechanism in Nd_6−*y*_WO_12−δ_ is expected to be largely controlled by the crystal structure, the conclusive determination of which is still lacking. This work presents a crystallographic study of Nd_5.8_WO_12−δ_ and molybdenum-substituted Nd_5.7_W_0.75_Mo_0.25_O_12−δ_ prepared by the citrate complexation route. High-resolution synchrotron and neutron powder diffraction data were used in combined Rietveld refinements to unravel the crystal structure of Nd_5.8_WO_12−δ_ and Nd_5.7_W_0.75_Mo_0.25_O_12−δ_. Both investigated samples crystallize in a defect fluorite crystal structure with space group *Fm* 3 *m* and doubled unit-cell parameter due to cation ordering. Mo replaces W at both Wyckoff sites 4*a* and 48*h* and is evenly distributed, in contrast with La_6−*y*_WO_12−δ_. X-ray absorption spectroscopy as a function of partial pressure *p*O_2_ in the near-edge regions excludes oxidation state changes of Nd (Nd^3+^) and W (W^6+^) in reducing conditions: the enhanced hydrogen permeation, *i.e.* ambipolar conduction, observed in Mo-substituted Nd_6−*y*_WO_12−δ_ is therefore explained by the higher Mo reducibility and the creation of additional – disordered – oxygen vacancies.

## Abstract 2

The crystal structures of non-substituted and Mo-substituted neodymium tungstates are described in detail through neutron diffraction and high-resolution X-ray diffraction. Combined X-ray and neutron diffraction refinements and electron probe micro-analysis were employed to locate Mo atoms in the crystal structure of Nd_6−*y*_W_1−*z*_Mo_*z*_O_12−δ_ (*z* = 0, 0.25), while X-ray absorption spectroscopy in the near-edge regions confirmed no changes in the oxidation states of Nd and W.

## 1. Introduction   

Catalytic membrane reactors (CMR), allowing simultaneous chemical reaction and product separation and improving thermodynamically limited reaction yields, are promising devices for process intensification owing to their compactness, simplicity and energy efficiency. In the past few years electro­chemically driven proton-conducting membranes have been employed in CMR for methane de­hydro­aromatization (Morejudo *et al.*, 2016 ▸) and steam methane reforming (Malerød-Fjeld *et al.*, 2017 ▸), resulting in higher yields, catalyst stability and separate high-purity H_2_ streams. Hydrogen production from water electrolysis has also been studied thoroughly (Choi *et al.*, 2019 ▸; Vøllestad *et al.*, 2019 ▸), as has ammonia production from water and N_2_ (Kyriakou *et al.*, 2020 ▸; Marnellos & Stoukides, 1998 ▸). In the above-mentioned cases, the employed membranes were based on BaZr_1−*x*−*y*_Ce_*x*_Y_*y*_O_3−δ_, a material that presents protonic and oxygen ionic conductivity (Ricote *et al.*, 2011 ▸, 2009 ▸; Katahira *et al.*, 2000 ▸; Choi *et al.*, 2018 ▸).

On the other hand, mixed protonic electronic conducting (MPEC) ceramics are selective to H_2_. This is due to protons being transported through their crystal structure, driven by a chemical potential gradient. The protons are charge compensated by transport of electrons and/or oxygen ions and do not need any external driving force. For this reason, MPEC ceramics are among the most promising candidates for gas separation, CMR (Deibert *et al.*, 2017 ▸) and protonic ceramic fuel cell technologies. Among different MPEC ceramics, rare earth tungsten oxides with the common formula RE_6_WO_12_ (RE = La, Nd) are presented as promising candidates due to their superior mixed conductivity and their ability to operate at high temperatures and pressures (Jordal *et al.*, 2004 ▸; Haugsrud, 2007 ▸).

Nd_6−*y*_WO_12−δ_ (NWO) and substituted NWO compounds have proved to be stable mechanically (Escolástico *et al.*, 2018 ▸) and chemically in reactive environments (CO_2_, CO, H_2_, H_2_O and H_2_S) (Escolástico *et al.*, 2015 ▸; Escolástico, Schroeder *et al.*, 2014 ▸), emerging as candidate materials for H_2_-permeable membranes in CMR or in gasification power plants.

The widely studied mixed conductors La_6−*y*_WO_12−δ_ (LWO) and substituted LWO crystallize in the fluorite-type crystal structure with a doubled cubic lattice due to the ordering of La and W (Scherb *et al.*, 2016 ▸; Fantin *et al.*, 2016 ▸, 2017 ▸, 2019 ▸; Magrasó *et al.*, 2009 ▸, 2012 ▸; Magrasó & Frontera, 2016 ▸; Kalland *et al.*, 2013 ▸). The unit cell can be best described with the formula La_28−*x*_W_4+*x*_O_54+3*x*/2_ν_2−3*x*/2_, where *x* is the amount of W^6+^ dissolving on the La2 site (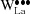 in Kröger–Vink notation) and ν the number of oxygen vacancies (Erdal *et al.*, 2012 ▸). A single-phase material sintered at *T* = 1773 K can be produced in the range 0.78 < *x* < 1.08, corresponding to an La/W ratio between 5.3 and 5.7 (Magrasó & Haugsrud, 2014 ▸). Seeger *et al.* (2013 ▸) found that the single-phase range depends on the sintering temperature and shifts to smaller La/W ratios at lower sintering temperature.

Oppositely to LWO (Seeger *et al.*, 2013 ▸), *A*-site doping (Escolástico, Schroeder *et al.*, 2014 ▸; Escolástico *et al.*, 2011 ▸; Haugsrud, 2007 ▸) improves the ambipolar conductivity of NWO and subsequently the permeation behaviour. *B*-site doping with Re (Escolástico, Somacescu & Serra, 2014 ▸; Escolastico *et al.*, 2013 ▸), U (Escolástico & Serra, 2015 ▸) or Mo (Escolastico *et al.*, 2013 ▸; Escolástico *et al.*, 2015 ▸; Ruf *et al.*, 2014 ▸; Vøllestad *et al.*, 2014 ▸; López-Vergara *et al.*, 2018 ▸) for W in both compounds further increases the total conductivity by one order of magnitude and, in the case of Nd_5.5_W_0.5_Mo_0.5_O_11.25−δ_, the H_2_ permeability by seven times compared with pure NWO at 1273 K (Escolástico *et al.*, 2017 ▸, 2015 ▸).

Little is known about the crystal structure of NWO and substituted NWO. Trunov (1968 ▸) and McCarthy *et al.* (1972 ▸) proposed in the late 1960s and early 1970s a tetragonal distorted or pseudo-tetragonal cell for Nd_6_WO_12_, although in both cases a few low-angle Bragg reflections were not indexed and were ascribed by the authors to superstructure reflections, ruling out possible secondary phases. More recent literature (Escolástico *et al.*, 2009 ▸, 2015 ▸) described the structure of NWO as cubic or tetragonal depending on the sintering temperature and composition. However, a conclusive structural description of pure and substituted NWO has not been presented so far. The goal of the present work was to elucidate the crystal structure of NWO and Mo-substituted NWO by combining synchrotron X-ray and neutron powder diffraction structural refinements. Since Escolástico *et al.* (2015 ▸) reported a phase transition for Nd_5.5_W_1−*z*_Mo_z_O_11.25−δ_ from cubic to rhombohedral at Mo substitution levels of around 50%, here a conservative substitution degree of 20% was chosen (*z* = 0.2). It was assumed that the stoichiometric differences in Nd content between the literature and specimen compositions were not relevant enough to induce any substantial deviation from the cubic crystal structure when *z* = 0.2, which was proved to be the case. The presented results will further support a comprehensive understanding of H_2_ transport within the NWO structure.

## 2. Experimental   

### 2.1. Sample synthesis and treatment   

Specimens were produced following a wet chemical route, the citrate complexation route (Seeger *et al.*, 2013 ▸; Escolástico *et al.*, 2009 ▸). With this synthesis method, it is possible to produce powders with high homogeneity, which is especially important for samples with a low concentration of substituents. Before the final sintering step the powders were uniaxially pressed into cylindrical discs and then sintered at 1773 K for 12 h in air with heating and cooling rates of 2 K min^−1^ to obtain single-phase materials with high crystallinity suitable for diffraction investigations. The sintered discs were polished to remove secondary phases (Nd_2_O_3_, Nd_10_W_2_O_21_) on the surface formed during sintering. Two different samples were produced: an undoped neodymium tungstate with a nominal composition of Nd_5.7_WO_12−δ_ (labelled NWO) and an Mo-substituted sample with a nominal composition of Nd_5.4_W_0.8_Mo_0.2_O_12−δ_ (labelled NWM). In order to remove adsorbed and/or absorbed water, the samples were dried under constant Ar or synthetic air flow at 1173 K for 4 h in a tubular furnace prior to the structural analysis. Deuteration was achieved by connecting two bubble bottles filled with D_2_O to the synthetic air stream at room temperature. The furnace was kept at 623 K for 4 h. Following heat treatments, the samples were transferred to the experimental stations in air-tight boxes filled with argon to prevent changes to the pre-treated specimens.

### 2.2. Compositional and thermogravimetric analysis   

Chemical composition was determined by electron-probe microanalysis (EPMA) using a JEOL JXA 8200 device. Wavelength-dispersive X-ray spectroscopy was carried out on 10 to 20 points measured on the surface of each grain and on cross sections of pieces of the sintered pellets to ensure homogeneous sampling of the powders. Only grains without visible pores and only obtained measurements with a calculated sum in mass percent of at least 95% were considered in the analysis. Elemental standards for tungsten, neodymium oxide and molybdenum oxide were used for the calibration, in order to increase the accuracy of the local composition measurements.

The mass loss of the pre-treated samples was determined during heating with a Netzsch TG209F1 Iris microbalance equipped with a mass spectrometer QMS 403 Aëolos with a heating ramp of 5 K min^−1^ and Ar atmosphere.

### 2.3. Phase and structural analysis   

Phase analysis was performed by Cu *K*α X-ray diffraction (XRD) using a Bruker D8 diffractometer in Bragg–Brentano geometry. Phase identification was aided by the International Centre for Diffraction Data (ICDD; http://www.icdd.com) PDF2 database within the *EVA14* software (Bruker, 2008 ▸) and Le Bail (Le Bail *et al.*, 1988 ▸) refinements of unit-cell parameters were performed using *TOPAS 4.2* (Evans, 2010 ▸; Coelho, 2018 ▸).

The crystal structure was resolved by combining neutron powder diffraction (ND) and high-resolution synchrotron X-ray powder diffraction (HRSXRD). Neutron diffraction was carried out on the fine-resolution powder diffractometer (FIREPOD) at the neutron reactor BERII at HZB (Helmholtz-Zentrum-Berlin, Germany) with wavelengths of 1.3084 (2), 1.7982 (1) and 2.8172 (2) Å (Franz & Hoser, 2017 ▸), using 6 mm diameter vanadium cans and measuring at room temperature. Neutrons are particularly useful for investigating rare earth tungstates since the difference in the coherent scattering lengths of the cations Nd [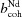 = 7.69 (5) fm], W [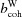 = 4.755 (18) fm] and Mo [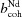 = 6.715 (20) fm] provide good contrast between them. Oxygen anions have a favourable neutron scattering length of 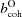 = 5.805 (4) fm.

HRSXRD measurements were performed on beamline ID22 (formerly ID31) (Fitch, 2004 ▸) at the European Synchrotron Radiation Facility (ESRF, Grenoble, France) for samples dried under Ar. A wavelength of 0.39987 (1) Å was used to minimize absorption effects. The powder samples were diluted with low-absorbing Al_2_O_3_ (NIST 676) which was used as internal standard and filled into polyimide capillaries of diameter 0.7 mm inside a glove bag. Further HRSXRD measurements for samples dried and deuterated under synthetic air were conducted on the material science (MS) beamline (Willmott *et al.*, 2013 ▸) at the Swiss Light Source (SLS). The MS beamline was equipped with a Mythen II microstrip detector (Gozzo *et al.*, 2010 ▸). The powder samples were loaded into glass capillaries of diameter 0.1 mm inside a glove box. To increase by 10–20% the contrast between the substituend Mo and the other cations Nd (56.6 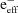) and W (67.48 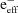), the wavelength was chosen just below the Mo *K* absorption edge at an energy of 19.9 keV [λ = 0.62284 (1) Å], reducing the effective number of electrons (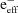) of Mo^6+^ from 36 e^−^ to 31.35 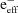. For further details on scattering power calculations and energy-dependent dispersion corrections the reader is referred to Cromer & Liberman (1981 ▸).

Combined Rietveld refinements, applying the X-ray and neutron diffraction pattern simultaneously, were performed using *GSAS II* (Toby & Von Dreele, 2013 ▸). The following refinement strategy was applied:

(i) The instrumental profile, wavelength and zero correction were refined using reference materials (HRSXRD: LaB_6_ NIST 660a and Si NIST 640d; neutron diffraction: Y_2_O_3_).

(ii) The background was fitted with a Chebychev polynomial function using 15 (BERII data), 21 (ESRF data) and/or 36 (SLS data) parameters and kept fixed until the last stage of the refinement, when it was refined together with the other structural parameters.

(iii) Unit-cell parameters and sample displacement parallel and perpendicular to the beam were refined together with the internal standard Al_2_O_3_. The internal standard was kept fixed to the tabulated values during refinement.

(iv) Finally, structural parameters (atomic displacement parameter ADP, site occupation factor SOF, fractional coordinates *x*, *y*, *z*) were refined (see *Results and discussion* [Sec sec3] section).

The quality of refinement was estimated by the weighted profile *R* factor *wR* and 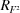 based on the structure factor *F_hkl_* of the crystallographic model (Toby, 2006 ▸; Rietveld, 1969 ▸).

### 2.4. XANES experiments   

XANES measurements at the W and Nd *L*_3_ absorption edges (10 207 and 6208 eV) were performed on the LISA beamline (BM-08) (d’Acapito *et al.*, 2019 ▸) at the ESRF. Samples were measured using a pair of Si(311) flat crystals; Si-coated focusing mirrors (*E*_cutoff_ ≃ 16 keV) were used for harmonic rejection. Measurements were performed on pellets (13 mm diameter) in transmission mode at room temperature; W and Cr standard foils were measured in transmission mode for energy calibration.

The spectra were acquired with a step of 5 eV in the pre-edge region Δ*E*_pe_ (*E*_0_ − 200 eV ≤ Δ*E*_pe_ ≤ *E*_0_ − 25 eV), a step of 0.025 eV in the XANES region (*E*_0_ − 25 eV < Δ*E*_XANES_ ≤ *E*_0_ + 25 eV) and a fixed *k* step of 0.03 Å^−1^ above *E*_0_ + 25 eV, up to maximum values of *k*_max_(Nd) = 11.6 Å^−1^ and *k*_max_(W) = 12 Å^−1^, for proper post-edge spectrum normalization.

Standard procedures (Lee *et al.*, 1981 ▸) were followed to extract the XANES signal: pre-edge background removal, spline modelling of bare atomic background, edge-step normalization using a polynomial function above the edge region, and energy calibration using the software *ATHENA* (Ravel & Newville, 2005 ▸).

## 3. Results and discussion   

### 3.1. Compositional and phase analysis   

**Figure 1:**
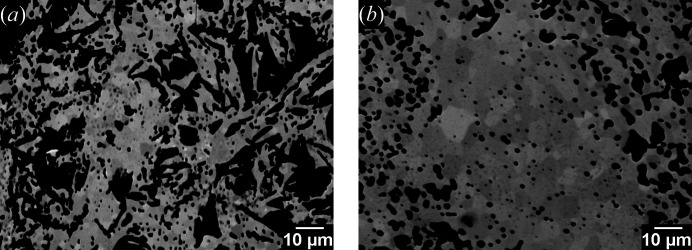
BSE micrographs of (*a*) NWO and (*b*) NWM.

Phase analysis of the as-synthesized powders performed by XRD and EPMA confirmed the single-phase composition of both samples. Table 1 ▸ lists the NWO and NWM compositions calculated from EPMA, together with the ratios Nd/(W + Mo) and Mo/(W + Mo). Fig. 1 ▸ shows backscattered electron (BSE) micrographs of specimens of NWO and NWM, where dark and light grey reflect the crystallographic orientation of the individual grains rather than a compositional gradient. No compositional differences between different grains and no secondary phases could be detected with EPMA.

**Figure 2:**
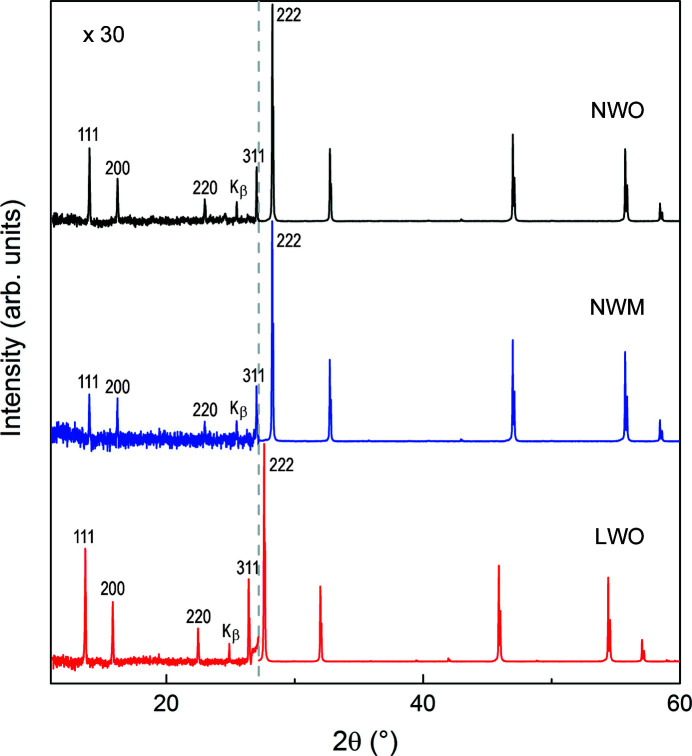
Powder XRD patterns for Nd_5.8_WO_12−δ_ (NWO), Nd_5.7_W_0.75_Mo_0.25_O_12−δ_ (NWM) and La_5.6_WO_12−δ_ (LWO). The *y* axis in the low-2θ region (10 ≤ 2θ ≤ 27.2°) is enlarged by a factor of 30 for better visualization.

Laboratory XRD data revealed a doubled fluorite cell consistent with the cell found for La_5.6_WO_12−δ_ (Scherb *et al.*, 2016 ▸; Fantin *et al.*, 2016 ▸; Magrasó *et al.*, 2012 ▸), with space group 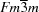 (No. 225). The smaller unit cell of NWO compared with LWO (see Fig. 2 ▸) reflects the smaller radius of Nd compared with La. Fluorite superstructure reflections, therefore indexed as 111 and 200 of the doubled cell, are weaker for the NWO and NWM samples than for the LWO sample. No secondary phases could be found within the detection limits of the diffractometer used.

### 3.2. Combined X-ray and neutron diffraction refinements on NWO   

**Figure 3:**
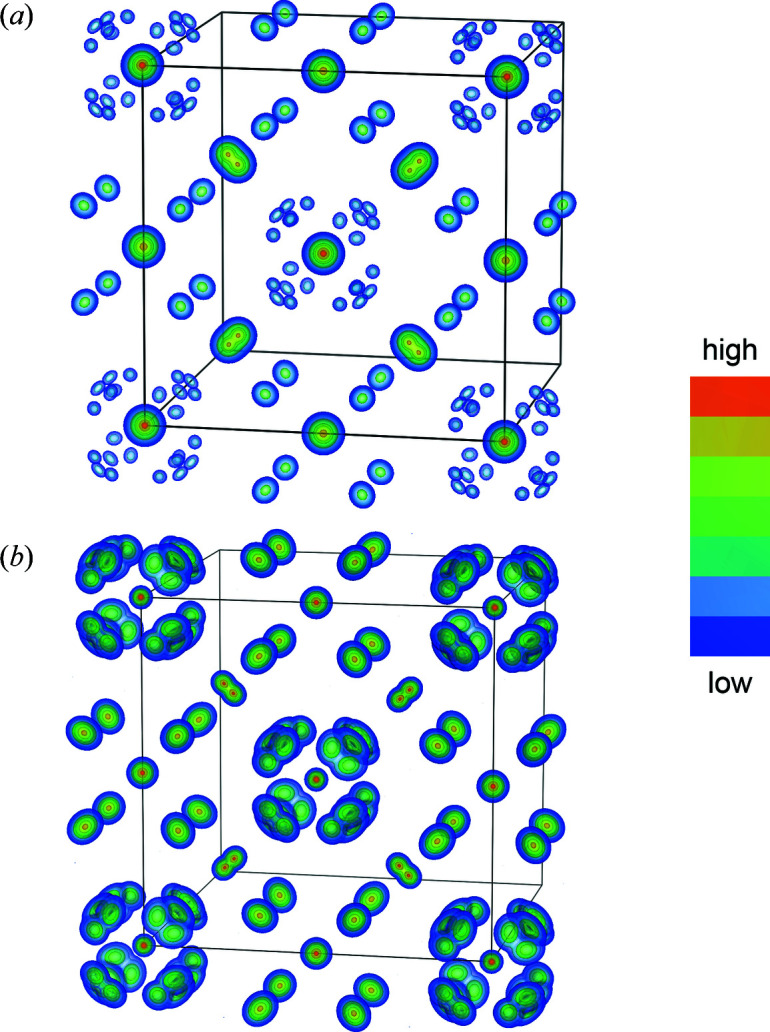
(*a*) The observed electron-density map calculated from HRSXRD and (*b*) the observed nuclear-density map calculated from ND at room temperature. Only the lattice plane (001) with the corresponding oxygen coordination is displayed. The colour scale was normalized to the maximal and minimal electron and nuclear density.

High-resolution XRD showed the absence of any tetragonal distortion of the fluorite structure, contrary to previous reports (McCarthy *et al.*, 1972 ▸; Trunov, 1968 ▸; Scherb, 2011 ▸), and the presence (3.6 wt%) of the secondary phase Nd_10_W_2_O_21_ (space group *Pbcn*, No. 60), which eluded both laboratory XRD and EPMA. The existence of this secondary phase can be explained by segregation of W-rich phases at the surface of the sintered discs, which also clarifies the difference between nominal and measured Nd, W and Mo content. The Nd_10_W_2_O_21_ phase was indexed and refined for the HRSXRD pattern with unit-cell parameters *a* = 16.416 (1) Å, *b* = 10.903 (1) Å and *c* = 10.929 (1) Å. However, due to the high number of reflections (6407) within the refined 2θ range of the HRSXRD data, Rietveld refinement with the main NWO phase and secondary Nd_10_W_2_O_21_ phase becomes excessively slow. Since the refinement of the main NWO phase is not influenced by the secondary Nd_10_W_2_O_21_ phase, the latter was not considered further for the combined X-ray and neutron refinements. Isosurface plots of the observed electron and nuclear scattering densities extracted from the HRSXRD and ND powder patterns (Fig. 3 ▸) show a splitting of the Wyckoff site 24*d* (0¼¼) in the [110] direction, away from one of the three mirror planes onto Wyckoff site 48*h* (0*yy*).

The split Wyckoff site 48*h* leads to two very near half-occupied Nd sites, in analogy to LWO, defining the locally disordered oxygen environment of the W1 site 4*a* and clearly visible in the nuclear-density map in Fig. 3 ▸(*b*). The oxygen site O1 is split from a regular arrangement on a cube on Wyckoff site 32*f* (*xxx*) onto 96*k* (*xxy*) by leaving the ternary axis along the mirror plane, observed already for LWO (Scherb *et al.*, 2016 ▸; Magrasó & Frontera, 2016 ▸). The disordered average crystal structure describing LWO was, therefore, used as a starting model for the Rietveld refinement of NWO. The defect nomenclature from LWO, according to Erdal *et al.* (2012 ▸) and Magrasó *et al.* (2012 ▸), can be adapted to NWO: its unit cell is described as Nd_28−*x*_W_4+*x*_O_54+3*x*/2_ν_2−3*x*/2_ with an *x* of 0.69 calculated from EPMA. The starting values for the cation SOFs were taken from EPMA and refined with chemical composition restraints to the results calculated from EPMA. Wyckoff site 4*a* with the highest electron density was occupied by W and site 4*b* by Nd. The remaining W (0.69 atoms) was located on the 48*h* site (Scherb *et al.*, 2016 ▸). In the first step of the structural refinement, the SOFs of the cation and anion sites were kept fixed. It is necessary to assume at least the site occupation for one site to remove direct correlations with the scale factor, since the SOFs contribute to the integrated area of the peaks. In complex materials like these defect fluorites, the form factor for each reflection is mostly a function of all atoms present in the system.

**Figure 4:**
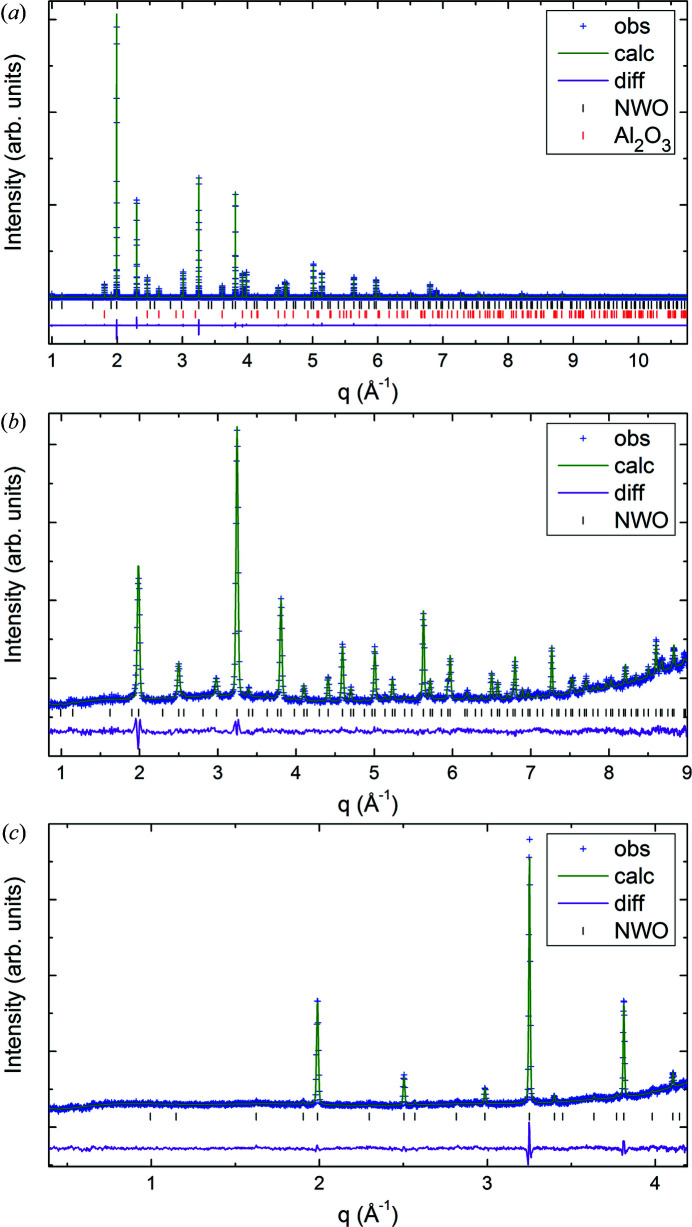
Final Rietveld plots of the combined refinements of (*a*) HRSXRD and (*b*), (*c*) ND data of (*b*) 1.31 Å and (*c*) 2.82 Å for NWO at 295 K. The patterns are plotted as a function of momentum transfer *q* = 4πsinθ/λ.

At first the ADPs were refined as isotropic while keeping the SOFs fixed. Then the fractional coordinates were refined, keeping SOFs and ADPs fixed. Then the ADPs were refined together with the coordinates and fixed SOFs. In the final cycles of the refinement, atomic anisotropic displacement parameters were used and refined together with the coordinates. This resulted in abnormally high ADPs for Nd1 on site 4*b* (see Table 2 ▸). An implementation of anti-site defects on both 4*a* and 4*b* Wyckoff sites and free refinement of the neutron data sets (no HRSXRD) with chemical restraints did not improve the refinement. In this case only the neutron patterns were used because of the better contrast between Nd and W. A subsequent combined refinement with the anti-site disorder fixed to the result from ND leads to even larger ADPs for the Nd1 site and smaller ADPs for the W1 site. Therefore, anti-site defects occupying the 4*a* and 4*b* Wyckoff sites can be ruled out. Closer inspection of the electron-density maps at the 4*b* site shows a remarkable disorder in the direction towards the 4*a* site. Although splitting the 4*b* (½½½) site into 1/6 occupied 24*e* (00*x*), where *x* is close to 0.5, reduced the ADPs for the Nd1 site, it did not improve the fit any further. Since the SOFs for the Nd1 and W1 sites refined to 1 (within the uncertainties), both SOFs were kept at 100% occupation for the rest of the analysis. Only the SOFs of the 48*h* and of the two oxygen Wyckoff sites were refined. Even this did not improve the fit or change the SOFs significantly from the starting values obtained from EPMA. Therefore, the SOFs for all cation sites were kept fixed during the final refinement and only the SOFs for O1 and O2 together with the ADPs and fractional coordinates were refined. The resulting atomic coordinates, displacement parameters, SOFs and residuals from the refinement are summarized in Table 2 ▸ and the corresponding Rietveld fits are shown in Fig. 4 ▸.

**Figure 5:**
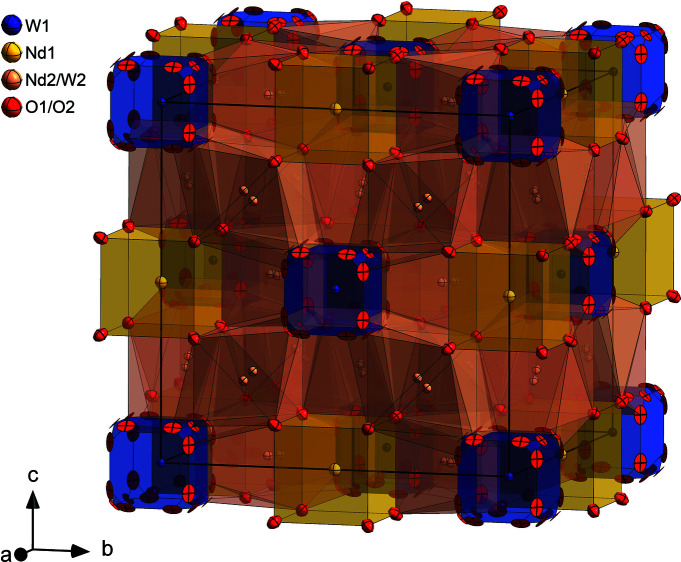
The disordered average crystal structure of NWO from combined Rietveld refinement to the HRSXRD and neutron diffraction data. Anisotropic ADPs are displayed at the 50% probability level.

The disordered average crystal structure for NWO, derived from a fluorite structure with a 2 × 2 × 2 supercell due to cation ordering, is plotted in Fig. 5 ▸. It is a face-centred cubic cell with W at the corners and face centres (Wyckoff site 4*a*), surrounded by the split O1 site, which is displaced from site 32*f* to 96*k* with 24 possible positions. This results in an average oxygen coordination of 5.6 (1). Nd occupies site 4*b* (edge centres) and is coordinated by O2 on site 32*f*, equivalent to 7.2 (1) oxygen neighbours arranged regularly on a cube. The higher ADPs for Nd on Wyckoff site 4*b* can be rationalized by local static disorder. In the disordered average picture the mixed occupied and split site 48*h* with an average oxygen coordination of 6.4 (1) is highly disordered, which can be better understood if the local arrangement is considered. On this site 0.7 cations out of 24 are occupied by W, which acts as a donor dopant (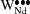 in Kröger–Vink notation), consuming oxygen vacancies and, hence, stabilizing the crystal structure. These 3% W anti-site defects on site 48*h* introduce further static displacements on a local length scale. The oxygen sublattice is also highly disordered, with a split O1 site and large ADPs for both oxygen sites. This is comparable to the LWO crystal structure, which can be therefore assumed as representative for NWO. The unit-cell composition was finally refined to Nd_27.3_W_4.7_O_51.2_.

**Figure 6:**
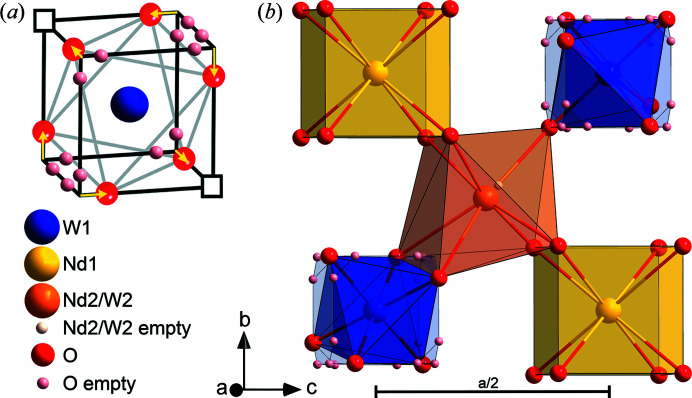
The local arrangement of NWO shown for one fluorite sub-cell layer. Two oxygen vacancies located on one diagonal of the cube coordinating W give order to the six remaining oxygens (*a*) and lead to a relaxation of Nd2/W2 in the direction of the edge-shared W octahedron (*b*).

The highly disordered average structure is described through local rearrangements and relaxation of both anions and cations. By analogy with LWO, in NWO an octahedral coordination for W can be assumed if two oxygen vacancies are placed on a diagonal of the cube and the remaining six oxygen ions relax in a direction towards the vacancies [see Fig. 6 ▸(*a*)], occupying one of the possible three positions of the disordered average model, *i.e.* the one next to the oxygen vacant site. The orientation of one W octahedron propagates ordering to the next octahedron via the split and half-occupied Nd2/W2 site [Fig. 6 ▸(*b*)]. In the local description, the Nd2/W2 site moves away from the defect and is closest and bonds to two oxygen ions of the WO_6_ octahedron.

### 3.3. HRSXRD refinement on Mo-substituted NWO   

The dry NWM sample was studied by HRSXRD to investigate the cation distribution and structural changes induced by the substitution of W by Mo. Small amounts (2.1 wt%) of the secondary phase Nd_10_W_2_O_21_ with space group *Pbcn* (No. 60) were found with HRSXRD and, as for NWO, it was no longer considered in the NWM structural refinements. The refined unit-cell parameters of this secondary phase, *a* = 16.425 (1) Å, *b* = 10.896 (1) Å and *c* = 10.935 (1) Å, are reported for completeness.

As the electron-density distribution for NWM did not deviate from that of NWO, the disordered NWO average structure was used as a starting model. At first, a refinement was performed for the single-atom model (*M*0), *i.e.* using only one atom per Wyckoff site in the NWO crystal structure (see Table 2 ▸). From the refined SOFs four different Mo distribution models were developed, in which 25% of Mo substituting W, determined with EPMA, have to be distributed in the crystal structure.

**Figure 7:**
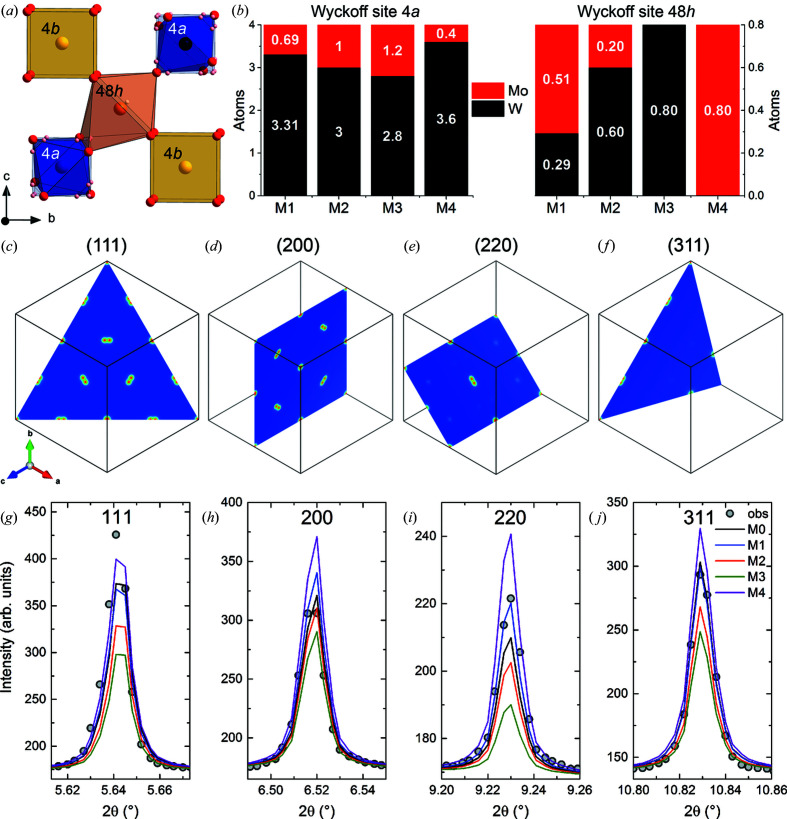
(*a*) The coordination of the cations, displayed for a half unit cell. (*b*) The different cation distribution models *M*1–*M*4 for the Wyckoff sites 4*a* and the split site 48*h*. (*c*)–(*f*) The electron density observed from HRSXRD, shown for (*c*) the (111), (*d*) the (200), (*e*) the (220) and (*f*) the (311) lattice planes. (*g*)–(*j*) The corresponding Rietveld fits to the superstructure reflections (*g*) 111, (*h*) 200, (*i*) 220 and (*j*) 311.

Comparing the ionic radii of six-coordinated W^6+^ (0.60 Å) and Mo^6+^ (0.59 Å) with that of eightfold Nd^3+^ (1.109 Å) (Shannon, 1976 ▸), it becomes apparent that Mo substitutes W on the W1 site 4*a* and on the mixed occupied and highly distorted Nd2/W2 site 48*h*. The Wyckoff site 4*b* occupied by Nd1 and coordinated in a regular cube by almost eight oxygens was not considered available for Mo in the fitted models. The single-atom model *M*0 and the four different models *M*1–*M*4 can be described as follows [see Figs. 7 ▸(*a*) and 7 ▸(*b*)]:

(i) *M*0: refinement performed with W on 4*a* and Nd on 4*b* and 48*h* sites and SOFs free (no Mo in the structure).

(ii) *M*1: SOFs for sites 4*a* and 48*h* calculated directly from the single-atom model.

(iii) *M*2: statistical distribution of Mo on both Wyckoff sites 4*a* and 48*h*.

(iv) *M*3: Mo only on 4*a* site (no Mo on 48*h* site).

(v) *M*4: W only on 4*a* site (no W on 48*h* site).

Models *M*1–*M*4 were refined keeping all *M*0-refined global parameters (instrumental profile, background and sample displacement) constant. The SOFs for the different distribution models, as listed in Table 3 ▸, were used and kept constant during the refinement. Only the scale factor, the fractional coordinates and ADPs for all atoms were refined. For better visualization, the number of Mo and W atoms on the two Wyckoff sites 4*a* and 48*h* are plotted for the models *M*1–*M*4 in Fig. 7 ▸(*b*).

Cation order is mainly reflected by the superstructure reflections already indexed as 111, 200, 220 and 311 of the double fluorite cell in the laboratory XRD patterns (Fig. 2 ▸). The weaker superstructure intensities shown by NWM relative to NWO (∼1:2 ratio) reflect the reduced scattering power of the Mo/W mixed sites 4*a*, 4*b* and 48*h*.

Closer inspection of the superstructure lattice planes (*hkl*) shown in Figs. 7 ▸(*c*)–7 ▸(*f*) and the corresponding fitted reflections Fig. 7 ▸(*g*)–7 ▸(*j*) reveals that the best fits occur for models *M*1 and *M*4. This is also reflected in the listed *R* values in Table 3 ▸. Indeed, when considering the effective number of electrons per site, models *M*2 and *M*3 show a lack of electrons on site 4*a*, which can be excluded on the basis of the larger *R* values reported in Table 3 ▸. Furthermore, in model *M*3, the effective numbers of electrons are equal for both sites 4*a* and 4*b*, differing only slightly from site 48*h* (56.6 to 57.0). Therefore, as no detectable cation order is observed, the superstructure lines in *M*3 are only weakly fitted. In *M*4 the superstructure reflections are over-fitted, related to the higher scattering power for the 4*a* site having the smallest amount of Mo and the highest amount of W. Taking the quality factors and careful inspection of the refinement results into account, *M*1 is proposed to fit the average crystal structure of NWM best. The substituted amount of Mo for W is more or less equally distributed over Wyckoff sites 4*a* and 48*h* (∼0.6 atoms per site) and not distributed proportionally to the W amount on each site proposed for Mo-substituted LWO. Fantin and co-workers found for Re- (Fantin *et al.*, 2016 ▸) and Mo-substituted (Fantin *et al.*, 2019 ▸) LWO a statistical distribution of the substituents over both W-containing sites, but Magrasó & Frontera (2016 ▸), on the other hand, found no clear preference for Mo substitution between the two W sites. A preference for Mo occupying the distorted and more flexible site 48*h* is found from the refinements to the different models. However, the uncertainties in the cation ratio and Mo concentration measured by EPMA prevent us from judging that fact categorically.

### 3.4. Combined X-ray and neutron diffraction refinements on NWM   

**Figure 8:**
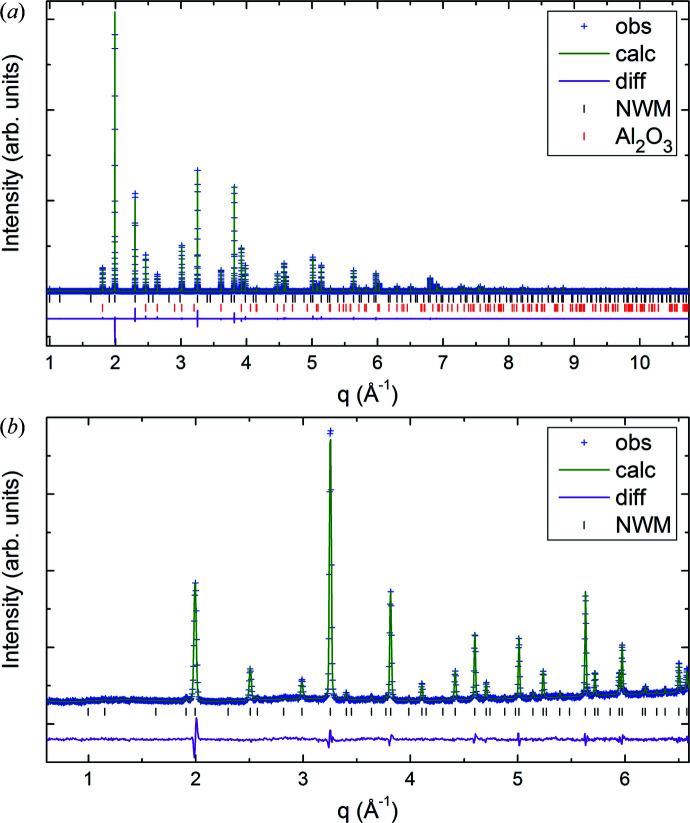
Final Rietveld plots for the combined refinements of (*a*) HRSXRD and (*b*) ND data for NWM at 295 K. The patterns are plotted as a function of the momentum transfer *q* = 4πsinθ/λ.

In order to validate the Mo distribution model *M*1 for the crystal structure of NWM, combined refinements of neutron and high-resolution X-ray data were performed. The combination of neutrons and X-rays has the advantage of opposite scattering contrast for Mo and W, increased for neutrons [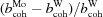 = 41%] and decreased for X-rays [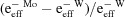 = −54%]. *M*1 was used as an initial model and the Rietveld refinement procedure was performed by analogy with NWO. In the final cycles of the refinement all coordinates and ADPs were refined together. The SOFs for Nd were kept fixed to the values obtained by EPMA and the SOFs for the other cations were refined freely, with the amount of Mo and W in the unit cell constrained to the results from EPMA. The quantity of Mo on the two W-containing sites was refined to 0.64 (2) Mo ions on site 4*a* and 0.57 (5) on site 48*h*. The details and results of the combined refinement are summarized in Table 4 ▸ and the corresponding Rietveld fits are plotted in Fig. 8 ▸. The refined SOFs are close to the ones used in model *M*1, which showed the best fit to the HRSXRD data collected close to the Mo *K* edge with increased scattering contrast for Mo ions. This leads to an almost even distribution of Mo on both W-containing Wyckoff sites. The crystal structure for NWM, except for small differences in the Nd/(W+Mo) ratio and in the SOFs for the oxygen sites, can be compared directly with the NWO structure depicted in Fig. 5 ▸.

### 3.5. Comparison of unit-cell parameters and bond lengths for dry and deuterated NWO and NWM   

**Figure 9:**
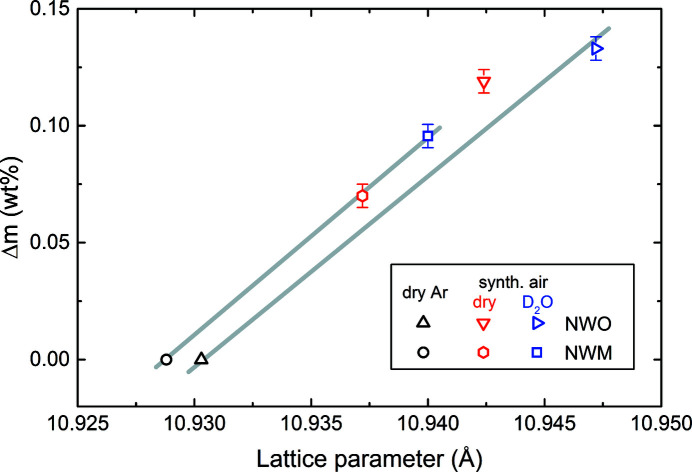
The mass gain of NWO and NWM, obtained from TG, displayed for different treatments as a function of the refined cubic lattice parameter *a*. The grey lines are guides for the eye.

Unit-cell parameters and bond lengths in NWO and NWM were investigated in relation to the partial pressures *p*(O_2_) and *p*(D_2_O) by annealing specimens under dry Ar, synthetic air and deuterated synthetic air. The neutron diffraction patterns showed negligible differences for deuterated and dry samples, due to the small amount of incorporated O or OD upon humidification. More interesting is the role of the cations, whose contrast is more visible with the HRSXRD measurements, presented and discussed in the following. The substitution of W by Mo leads to a decrease in the unit-cell parameter for NWM, rationalized by the smaller ionic radius of Mo^6+^ (0.59 Å) compared with that of W^6+^ (0.60 Å). However, Nd is the largest element in the compound. The Nd content, and in this specific case the Nd/(W + Mo) ratio, plays an important role and must be considered for the unit-cell parameter discussion. Indeed, the cationic W/Mo anti-site defects on Nd2 site 48*h* are directly determined by the *x* values in Nd_28−*x*_(W_1−*z*_Mo_*z*_)_4+*x*_O_54+3*x*/2_ν_2−3*x*/2_. Having a larger ratio for NWO (Nd/W = 5.82, *i.e.* *x* = 0.69) than for NWM [Nd/(W + Mo) = 5.68, *i.e.* *x* = 0.79] corresponds to more Nd^3+^ (*r*_ion_ = 1.109 Å) ions in the crystal structure and thus to a smaller number of 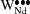 anti-site defects in NWO, leading to a larger unit-cell parameter than in NWM. The NWO and NWM unit-cell parameters depend on the *p*(O_2_) and *p*(D_2_O) partial pressures as well, due to filling of the oxygen vacancies with O and OD, respectively. This is described in Fig. 9 ▸, where the mass uptake Δ*m* obtained from thermogravimetric analysis (TG) is shown as a function of the corresponding unit-cell parameter, hinting at direct proportionality between *p*(O_2_)/*p*(D_2_O) and unit-cell size.

The larger increase in unit-cell parameter for NWO with increasing *p*(O_2_) and *p*(D_2_O) can be explained by the higher Nd/W ratio and hence higher number of oxygen vacancies that can be filled. From Fig. 9 ▸, one can then conclude that sample oxidation, *i.e.* oxygen vacancy filling, is more relevant for unit-cell expansion than the partial substitution of W by Mo. On the other hand, the small unit-cell parameter difference between the two NWM and NWO reduced specimens (dry Ar) is given by the lower Nd content in NWM combined with the partial reduction of Mo^6+^ to Mo^5+^ and/or Mo^4+^, *i.e.* by the larger ionic radius of reduced Mo ions (see Table 5 ▸).

The cation–oxygen bond distances for the different treatments, obtained from Rietveld refinements, are listed in Table 6 ▸. From the cation side, the larger bond distances for the W1/Mo1—O1 bonds in NWM compared with NWO can be correlated with the higher reducibility, and thus larger sizes, of Mo compared with W. A higher reducibility for Mo in a 50% Mo-substituted NWM was indeed found by Escolástico *et al.* (2015 ▸) using X-ray photoelectron spectroscopy analysis: the oxidation state of Mo under reducing conditions changed from Mo^6+^ to Mo^4+^. The larger Nd1—O2 bond distance for NWM is attributed to the higher coordination number of Nd1 in NWM than in NWO. The average Nd2—O bond distance is increased for NWO, which can be directly related to the higher Nd/(W + Mo) ratio of NWO compared with NWM, where a higher Nd/(W + Mo) ratio equals more Nd atoms (largest ionic radii) on Wyckoff site 48*h*. From the anion side, the filling of oxygen vacancies, *i.e.* an increase in *p*(O_2_)/*p*(D_2_O), leads to an increase in coordination number and a larger cation ionic radius (see Table 5 ▸), and hence larger bond distances and unit cell (see Fig. 9 ▸). This holds true for the W1/Mo1—O1 bonds. However, the Nd1—O2 bonds seem to behave in the opposite way. Several factors should be considered to account for this, namely the shared nature of O2 atoms between Nd1 and Nd2, the simultaneous expansion of Nd2—O2 bond lengths in the local sevenfold polyhedra, and the Wyckoff-site relative amount of Nd1 (four atoms) and Nd2 (*ca* 23 atoms). The large difference in the Nd1—O2 bond distance could also be correlated to the space group used for the refinements, as the oxygen site O2 on 32*f* (*xxx*) can only move in the direction of the Nd1—O2 bonds and cannot relax in the dry Ar state. This interpretation would also explain the large ADPs found for the O2 and Nd1 sites, though it is likely that the decrease in the Nd1—O2 bond length with increasing *p*(O_2_)/*p*(D_2_O) is a function of all the above-mentioned factors, and a final statement cannot be provided. The partial reduction of Nd^3+^ to Nd^2+^ under dry Ar is, however, excluded by the X-ray absorption spectroscopy studies reported in the following section, corroborated by the findings of Escolástico *et al.* (2015 ▸) under even more reducing conditions (dry Ar/H_2_).

### 3.6. XANES on NWO and NWM   

**Figure 10:**
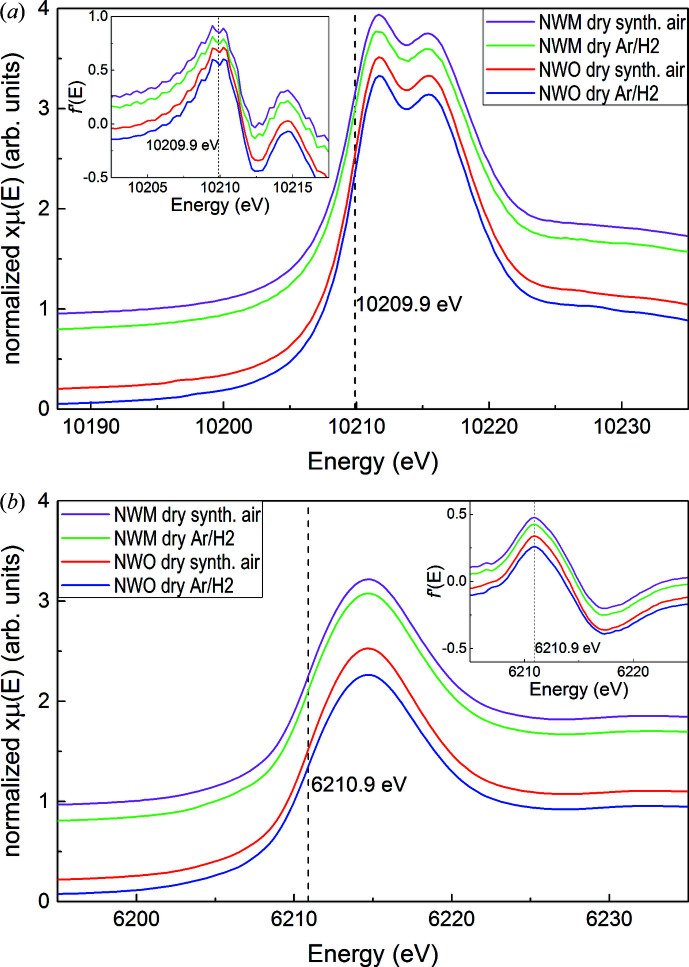
XANES spectra for oxidized and reduced NWO and NWM at (*a*) the W and (*b*) the Nd *L*_3_ absorption edge. The insets show the first derivatives of the normalized absorption spectra.

Through XANES measurements one investigates the oxidation state, bonding environment and local geometry (Henderson *et al.*, 2014 ▸) around a selected element in a given compound. The selectivity of the angular momentum allows, in addition, the probing of well defined final states. By measuring the *L*_3_ edges of Nd and W in reduced and oxidized NWO and NWM, the transitions from 2*p*_3/2_ to vacant *s* and *d* orbitals are probed. As the *p*→*d* transition contribution is about 50 times larger than that of the *p*→*s* transition (Teo & Lee, 1979 ▸), it is generally assumed that the probed final states are mainly unoccupied *d* states. In Fig. 10 ▸, the *L*_3_-edge spectra of W [Fig. 10 ▸(*a*)] and Nd [Fig. 10 ▸(*b*)] in dry Ar/H_2_ (reducing atmosphere) and dry synthetic air (oxidizing atmosphere) NWO and NWM specimens are presented.

From Fig. 10 ▸, the first information retrieved is the oxidation states of Nd and W in NWO and NWM. No change in the peak position is observed between the different pre-treatments at the Nd and W edges, either in NWO or in NWM. All the first peak maxima of the first derivatives (*cf.* inset) at the W edges, corresponding to the main absorption edge at 10209.90 (5) eV marked in Fig. 10 ▸(*a*), lie well below 0.05 eV, which is taken as a strict reasonable limit above which meaningful differences may occur. It is recalled that a change in oxidation state would produce shifts of the order of a few electronvolts. Similarly, all the peak maxima of the first derivatives at the Nd *L*_3_ edge are found to lie at 6210.90 (5) eV. The oxidation states of both Nd and W are, therefore, independent of the pre-treatment used and of Mo concentration, *i.e.* Nd and W preserve their +3 and +6 oxidation states, respectively, even in highly reducing atmospheres such as dry Ar/H_2_.

Secondly, the bonding environment is addressed. From Fig. 6 ▸, it is clear that ligand fields due to oxygen atoms act on both Nd (Nd1, Nd2) and W (W1, W2) sites. The relation between the Nd *L*_3_ edge and XANES is not easy to interpret, as the ligand field is the sum of a sevenfold oxygen coordination (Nd on 48*h*, ∼85%) and an eightfold coordination (Nd on 4*b*, ∼15%). Asakura *et al.* (2014 ▸) observed a linear relation between the FWHM of Nd-based compound *L*_3_ edges and coordination number, where, to a first approximation, a larger FWHM corresponds to lower coordination. From Fig. 10 ▸(*b*), a larger FWHM for NWO dry Ar/H_2_ [8.3 (3) eV] compared with that for NWO dry synthetic air [7.4 (3) eV] hints at a larger oxygen loss in NWO dry Ar/H_2_, which in turn decreases the average Nd—O coordination. A slight change in the FWHM is seen in NWM as well [dry Ar/H_2_: FWHM = 8.2 (3) eV; dry synthetic air: FWHM = 7.9 (3) eV], which agrees with the fact that water uptake in NWM is about a factor of two less than that of NWO (*cf.* Fig. 9 ▸). The absolute FWHM values at the Nd *L*_3_ edge are close to those found in Nd-based oxide compounds, where Nd is seven-coordinated (Asakura *et al.*, 2014 ▸). Specifically, the reported seven-coordinated Nd shows FWHMs of about 7 eV in Nd_4_CuO_7_ and Nd_4_PdO_7_. However, further systematic studies of NWO local structures and bond-length analyses must be carried out. For this reason, the Nd-bonding environment and its local coordination is not discussed further here.

The average W—O coordination number is the result of a majority of octahedral symmetry (∼80%, W on 4*a*) and a minority of six- or sevenfold symmetry (∼20%, W on 48*h*). In Fig. 10 ▸(*a*), a clear split of the W *L*_3_-edge white line of about Δ*E*_cf_ = 3.7 eV (cf stands for crystal field) is observed. The split is the consequence of the crystal field which acts on W, splitting the 5*d* orbital into the *t*_2*g*_ and *e*_*g*_ components, with Δ*E*_cf_ = 3.7 eV their energy difference. Δ*E*_cf_ is commonly referred to as 10Dq and it is a measure of how strong the ligand crystal field is. Different information on the point-group symmetry and orbital overlapping of W in NWO can be inferred from the 10Dq value when compared with the literature. It is reported that the higher the 10Dq value, the less distorted the *O_h_* symmetry of a six-coordinate environment (Yamazoe *et al.*, 2008 ▸), where 10Dq values of 4.9 and 5.6 eV were observed for pure octahedral symmetry (*O_h_*, Ba_2_NiWO_6_) and nearly octahedral symmetry (*D*_2_, Cr_2_WO_6_), respectively (Yamazoe *et al.*, 2008 ▸). A value of 10Dq = 4.8 (3) eV is observed at the W *L*_3_ edge in two different non-substituted LWO specimens (not shown here). The 10Dq value for LWO is slightly higher than the corresponding value for NWO, hinting at a more regular *O_h_*-like symmetry in the former. A similar 10Dq value to NWO is found for WO_3_ (10Dq = 4.0 eV) which presents a more distorted octahedral symmetry.

In yttria-stabilized zirconia (YSZ), which shows a fluorite structure like NWO, it is reported that the displacive cubic to tetragonal transition is driven by the 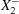 phonon soft modes of oxygen ions (Schelling *et al.*, 2001 ▸), whose large vibrational amplitudes and low-frequency dynamics are due to an almost flat, highly anharmonic potential well. It is known that structurally disordered ionic conductors such as YSZ and NWO or LWO (Fantin *et al.*, 2016 ▸) are instrinsically anharmonic, or at least they present a mobile sublattice, in this case the O1 oxygen atoms bonded to W1. The analogy between the low-frequency dynamics of the oxygen ions in YSZ and the O1 oxygen ions in the LWO and NWO crystal structure is the starting point for a deeper comprehension of the decreasing symmetry of Ln_6−*x*_WO_12−δ_ with decreasing Ln ionic radius (McCarthy *et al.*, 1972 ▸).

## 4. Conclusions   

The structural effects on the defect fluorite-type crystal structure caused by the complete substitution of Nd on the *A* site of La_6−*x*_WO_12−δ_ and the partial substitution of the *B* site of Nd_6−*x*_WO_12−δ_ by Mo were studied by combined neutron and high-resolution synchrotron powder diffraction. The crystal structure is characterized by the following: (i) a defect fluorite structure (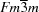) with unit-cell doubling caused by cation order, analogous to La_6−*x*_WO_12−δ_ (Scherb *et al.*, 2016 ▸); (ii) Wyckoff sites 4*a*, 4*b* and split 24*g* in the [110] direction, fully occupied by W and Nd and half-occupied by Nd and W, respectively; and (iii) a highly disordered anion sublattice, which enables excellent transport properties, as reported in the literature (Escolástico *et al.*, 2017 ▸, 2015 ▸). In addition, in the NWM structure, (iv) substituting Mo for W does not change the doubled fluorite crystal structure found for NWO; (v) Mo occupies both W sites, 4*a* and 48*h*; and (vi) no ordering of oxygen vacancies is observed, crucially, since proton mobility strongly inversely correlates with oxygen vacancy ordering. Unlike Mo- (Fantin *et al.*, 2019 ▸) and Re-substituted (Fantin *et al.*, 2016 ▸) LWO, the distribution of substituted (Mo) ions on 4*a* and 48*h* is roughly equal, as proven by refining different distribution models in combined neutron and X-ray refinements. As XANES shows, Nd^3+^ and W^6+^ keep their oxidation states in reducing conditions. Therefore, in reducing conditions, Mo changes its oxidation state at least partially from Mo^6+^ to Mo^4+^ (Escolástico *et al.*, 2015 ▸) as suggested by the combination of diffraction and thermogravimetry results. Therefore, the enhanced hydrogen permeation for the Mo-substituted samples can be attributed to the higher *n*-type conductivity for NWM which is related to the higher reducibility of Mo compared with W. Creating additional oxygen vacancies – in a disordered sublattice – and electrons leads to an increase in H_2_ flow for Mo-substituted NWO over the whole temperature range compared with unsubstituted NWO (Escolástico *et al.*, 2015 ▸, 2017 ▸; Li *et al.*, 2015 ▸). Such high ambipolar conductivity and outstanding hydrogen flow in a chemically stable structure show the high potential for separation techniques under reducing conditions.

## Supplementary Material

Crystal structure: contains datablock(s) NWO_dry_Ar_X+N_final_3_publ, NWO_dry_Ar_X+N_final_3_overall, NWO_dry_Ar_X+N_final_3_phase_1, NWO_dry_Ar_X+N_final_3_phase_0, NWO_dry_Ar_X+N_final_3_pwd_0, NWO_dry_Ar_X+N_final_3_pwd_1, NWO_dry_Ar_X+N_final_3_pwd_2. DOI: 10.1107/S1600576720012698/kc5116sup1.cif

## Figures and Tables

**Table 1:** Results from EPMA, nominal (at.%) cation ratios and composition for NWO and NWM

	NWO		NWM	
Label	Nominal	EPMA	Nominal	EPMA
Nd/(W + Mo)	5.7	5.82 (9)	5.4	5.68 (11)
Mo/(W + Mo)	0	0	0.2	0.25 (2)
Composition	Nd_5.82_WO_12−δ_		Nd_5.68_W_0.75_Mo_0.25_O_12−δ_	
Secondary phase	No		No	

**Table:** The coordinates, SOFs and ADPs are plotted for the combined X-ray and neutron refinement for NWO treated under dry Ar, labelled with an asterisk (combined refinement with weight factor 0.1:10:20). The ADPs *U_ij_* are multiplied by 100.

Treatment	Instrument	λ (Å)	Observations	Reflections	*R* (mm)	μ*R*	*wR* (%)	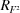 (%)	*a* (Å)	*V* (Å^3^)
Dry Ar*	ID22, ESRF	0.39987 (1)	36501	217	0.35	0.70	7.42*	3.67	10.9303 (1)	1305.86 (1)
Dry Ar*	E9, HZB	1.3084 (2)	1735	137	3	0.18	8.43*	8.68		
Dry Ar*	E9, HZB	2.8172 (2)	1735	22	3	0.35	20.65*	7.55		
Synthetic air	MS, SLS	0.62284 (1)	18334	265	0.05	0.74	2.55	2.69	10.9424 (1)	1310.20 (1)
D_2_O in synthetic air	MS, SLS	0.62284 (1)	18334	265	0.05	0.74	3.04	2.91	10.9472 (1)	1311.93 (1)

**Table:** 

Atom	Site	*x*	*y*	*z*	SOF	*U*_11_ (Å^2^)	*U*_22_ (Å^2^)	*U*_33_ (Å^2^)	*U*_12_ (Å^2^)	*U*_13_ (Å^2^)	*U*_23_ (Å^2^)
W1	4*a*	0	0	0	1.0	0.729 (35)	0.756 (6)	0.756 (6)	0	0	0
Nd1	4*b*	0.5	0.5	0.5	1.0	1.419 (59)	1.571 (7)	1.571 (7)	0	0	0
Nd2	48*h*	0	0.2365 (1)	0.2365 (1)	0.486	0.714 (60)	0.851 (45)	0.851 (45)	0	0	0.034 (48)
W2	48*h*	0	0.2365 (1)	0.2365 (1)	0.014	0.714 (60)	0.851 (45)	0.851 (45)	0	0	0.034 (48)
O1	96*k*	0.1132 (4)	0.1132 (4)	0.0668 (6)	0.233 (4)	0.92 (33)	0.92 (33)	3.26 (42)	−0.88 (24)	−0.16 (24)	−0.16 (24)
O2	32*f*	0.3658 (2)	0.3658 (2)	0.3658 (2)	0.900 (9)	1.73 (10)	1.73 (10)	1.73 (10)	0.33 (13)	0.33 (13)	0.33 (13)

**Table 3:** Cation distribution models for Mo-substituted NWO in space group 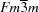 The Wyckoff site 48*h* is split and has a total occupancy of 0.5. The weighted profile *R* factor *wR* and the 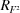 for full pattern Rietveld refinements are also listed.

Model	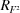	*wR*	Wyckoff site	SOF(W)	SOF(Mo)	SOF(Nd)
*M*0	2.76	2.54	4*a* (000)	0.907 (2)	–	–
			48*h* (0*yy*)	–	–	0.505 (1)

*M*1	2.61	2.54	4*a* (000)	0.8263	0.1737	–
			48*h* (0*yy*)	0.0061	0.0105	0.4833

*M*2	3.68	2.58	4*a* (000)	0.75	0.25	–
			48*h* (0*yy*)	0.0125	0.0042	0.4833

*M*3	4.45	2.67	4*a* (000)	0.70	0.30	–
			48*h* (0*yy*)	0.0167	–	0.4833

*M*4	1.92	2.64	4*a* (000)	0.90	0.10	–
			48*h* (0*yy*)	–	0.0167	0.4833

**Table:** The coordinates, SOFs and ADPs are plotted for the combined X-ray and neutron refinement for NWM treated under dry Ar, labelled with an asterisk (combined refinement with weight factor 1:10). The anisotropic displacement parameters *U_ij_* are multiplied by 100.

Treatment	Instrument	λ (Å)	Observations	Reflections	*R* (mm)	μ*R*	*wR* (%)	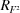 (%)	*a* (Å)	*V* (Å^3^)
Dry Ar*	ID22, ESRF	0.39987 (1)	36501	217	0.35	0.56	6.70*	3.97	10.9288 (1)	1305.32 (1)
Dry Ar*	E9, HZB	1.7982 (1)	1753	62	3	0.24	12.28*	4.76		
Synthetic air	MS, SLS	0.62284 (1)	18334	265	0.05	0.74	2.54	2.61	10.9372 (1)	1308.33 (1)
D_2_O in synthetic air	MS, SLS	0.62284 (1)	18334	265	0.05	0.74	2.53	2.55	10.9400 (1)	1309.34 (1)

**Table:** 

Atom	Site	*x*	*y*	*z*	SOF	*U*_11_ (Å^2^)	*U*_22_ (Å^2^)	*U*_33_ (Å^2^)	*U*_12_ (Å^2^)	*U*_13_ (Å^2^)	*U*_23_ (Å^2^)
W1	4*a*	0	0	0	0. 841 (6)	0.836 (30)	0.836 (30)	0.836 (30)	0	0	0
Mo1	4*a*	0	0	0	0.159 (6)	0.836 (30)	0.836 (30)	0.836 (30)	0	0	0
Nd1	4*b*	0.5	0.5	0.5	1.0	1.410 (41)	1.410 (41)	1.410 (41)	0	0	0
Nd2	48*h*	0	0.2365 (1)	0.2365 (1)	0.483	0.661 (42)	0.795 (29)	0.795 (29)	0	0	0.089 (31)
W2	48*h*	0	0.2365 (1)	0.2365 (1)	0.005 (1)	0.661 (42)	0.795 (29)	0.795 (29)	0	0	0.089 (31)
Mo2	48*h*	0	0.2365 (1)	0.2365 (1)	0.012 (1)	0.661 (42)	0.795 (29)	0.795 (29)	0	0	0.089 (31)
O1	96*k*	0.1141 (3)	0.1141 (3)	0.0676 (4)	0.239 (3)	0.78 (21)	0.78 (21)	4.31 (30)	−1.27 (14)	−0.85 (17)	−0.85 (17)
O2	32*f*	0.3652 (2)	0.3652 (2)	0.3652 (2)	0.923 (7)	1.950 (62)	1.950 (62)	1.950 (62)	0.245 (73)	0.245 (73)	0.245 (73)

**Table 5:** Ionic radii for different coordination according to Shannon (1976 ▸)

		Ionic radii (Å) for coordination				
Element	Valence	4	5	6	7	8
Nd	+2				1.230†	1.290
	+3			0.983	1.046†	1.109

W	+4			0.660		
	+5			0.620		
	+6	0.420	0.510	0.600	0.690†	

Mo	+3			0.690		
	+4			0.650		
	+5	0.460		0.610		
	+6	0.410	0.500	0.590	0.730	

O	−2	1.380		1.400		1.420

† Interpolated from tabulated data.

**Table 6:** Cation–oxygen bond lengths for the disordered average and the local model for NWO and NWM from Rietveld refinements for treatments under dry Ar, synthetic air and deuterated synthetic air The number of bonds in the disordered average and the local model was ascertained for full site occupation.

	Coordination		NWO			NWM		
Bond length (Å)	Average	Local	Dry Ar	Synthetic air	D_2_O in synthetic air	Dry Ar	Synthetic air	D_2_O in synthetic air
W1—O1	24×	6×	1.895 (4)	1.924 (2)	1.931 (3)	1.912 (2)	1.937 (2)	1.938 (2)
Nd1—O2	8×	8×	2.540 (2)	2.529 (1)	2.527 (1)	2.551 (1)	2.534 (1)	2.540 (1)
Nd2—O1	2×	0×	2.042 (4)	2.019 (3)	2.011 (3)	2.031 (3)	2.007 (3)	2.003 (2)
Nd2—O2	4×	4×	2.324 (2)	2.330 (1)	2.332 (1)	2.320 (1)	2.327 (1)	2.327 (1)
Nd2—O1	4×	2×	2.436 (4)	2.414 (2)	2.406 (3)	2.425 (3)	2.403 (3)	2.400 (2)
Nd2—O1	2×	1×	2.605 (6)	2.606 (4)	2.608 (4)	2.598 (4)	2.600 (3)	2.603 (3)
Nd2—O1	4×	0×	2.975 (6)	2.975 (4)	2.977 (4)	2.967 (3)	2.970 (3)	2.974 (3)
Nd2—O	16×		2.515 (4)	2.508 (3)	2.506 (3)	2.507 (3)	2.501 (3)	2.501 (2)
Nd2—O		7×	2.396 (3)	2.393 (2)	2.393 (2)	2.390 (2)	2.388 (2)	2.387 (2)

## References

[bb1] Acapito, F. d’, Lepore, G. O., Puri, A., Laloni, A., La Manna, F., Dettona, E., De Luisa, A. & Martin, A. (2019). *J. Synchrotron Rad.* **26**, 551–558. 10.1107/S160057751801843X 30855267

[bb2] Asakura, H., Shishido, T., Fuchi, S., Teramura, K. & Tanaka, T. (2014). *J. Phys. Chem. C*, **118**, 20881–20888.

[bb3] Bruker (2008). *Diffracplus Evaluation Package EVA 14*. Release 15 July 2008. Bruker AXS GmbH, Karlsruhe, Germany.

[bb61] Coelho, A. A. (2018). *J. Appl. Cryst.* **51**, 210–218.

[bb4] Choi, S., Davenport, T. C. & Haile, S. M. (2019). *Energy Environ. Sci.* **12**, 206–215.

[bb5] Choi, S., Kucharczyk, C. J., Liang, Y. G., Zhang, X. H., Takeuchi, I., Ji, H. I. & Haile, S. M. (2018). *Nat. Energ.* **3**, 202–210.

[bb6] Cromer, D. T. & Liberman, D. A. (1981). *Acta Cryst.* A**37**, 267–268.

[bb7] Deibert, W., Ivanova, M. E., Baumann, S., Guillon, O. & Meulenberg, W. A. (2017). *J. Membr. Sci.* **543**, 79–97.

[bb8] Erdal, S., Kalland, L. E., Hancke, R., Polfus, J., Haugsrud, R., Norby, T. & Magrasó, A. (2012). *Int. J. Hydrogen Energy*, **37**, 8051–8055.

[bb9] Escolástico, S., Schroeder, M. & Serra, J. M. (2014). *J. Mater. Chem. A*, **2**, 6616–6630.

[bb10] Escolastico, S., Seeger, J., Roitsch, S., Ivanova, M., Meulenberg, W. A. & Serra, J. M. (2013). *ChemSusChem*, **6**, 1523–1532. 10.1002/cssc.201300091 23828818

[bb11] Escolástico, S. & Serra, J. M. (2015). *J. Membr. Sci.* **489**, 112–118.

[bb12] Escolástico, S., Solís, C., Haugsrud, R., Magrasó, A. & Serra, J. M. (2017). *Int. J. Hydrogen Energy*, **42**, 11392–11399.

[bb13] Escolástico, S., Solís, C. & Serra, J. M. (2011). *Int. J. Hydrogen Energy*, **36**, 11946–11954.

[bb14] Escolástico, S., Somacescu, S. & Serra, J. M. (2014). *Chem. Mater.* **26**, 982–992.

[bb15] Escolástico, S., Somacescu, S. & Serra, J. M. (2015). *J. Mater. Chem. A*, **3**, 719–731.

[bb16] Escolástico, S., Stournari, V., Malzbender, J., Haas-Santo, K., Dittmeyer, R. & Serra, J. M. (2018). *Int. J. Hydrogen Energy*, **43**, 8342–8354.

[bb17] Escolástico, S., Vert, V. B. & Serra, J. M. (2009). *Chem. Mater.* **21**, 3079–3089.

[bb18] Evans, J. S. O. (2010). *Mater. Sci. Forum*, **651**, 1–9.

[bb19] Fantin, A., Scherb, T., Seeger, J., Schumacher, G., Gerhards, U., Ivanova, M. E., Meulenberg, W. A., Dittmeyer, R. & Banhart, J. (2016). *J. Appl. Cryst.* **49**, 1544–1560.

[bb20] Fantin, A., Scherb, T., Seeger, J., Schumacher, G., Gerhards, U., Ivanova, M. E., Meulenberg, W. A., Dittmeyer, R. & Banhart, J. (2017). *Solid State Ionics*, **306**, 104–111.

[bb21] Fantin, A., Scherb, T., Seeger, J., Schumacher, G., Gerhards, U., Ivanova, M. E., Meulenberg, W. A., Dittmeyer, R. & Banhart, J. (2019). *J. Appl. Cryst.* **52**, 1043–1053.

[bb22] Fitch, A. N. (2004). *J. Res. Natl Inst. Stand. Technol.* **109**, 133–142. 10.6028/jres.109.010 PMC4849623 27366602

[bb23] Franz, A. & Hoser, A. (2017). *J. Large-Scale Res. Facil.* **3**, A103.

[bb24] Gozzo, F., Cervellino, A., Leoni, M., Scardi, P., Bergamaschi, A. & Schmitt, B. (2010). *Z. Kristallogr. Cryst. Mater*, **225**, 616.

[bb25] Haugsrud, R. (2007). *Solid State Ionics*, **178**, 555–560.

[bb26] Henderson, G. S., de Groot, F. M. F. & Moulton, B. J. A. (2014). *Rev. Mineral. Geochem.* **78**, 75–138.

[bb27] Jordal, K., Bredesen, R., Kvamsdal, H. M. & Bolland, O. (2004). *Energy*, **29**, 1269–1278.

[bb28] Kalland, L.-E., Magrasó, A., Mancini, A., Tealdi, C. & Malavasi, L. (2013). *Chem. Mater.* **25**, 2378–2384.

[bb29] Katahira, K., Kohchi, Y., Shimura, T. & Iwahara, H. (2000). *Solid State Ionics*, **138**, 91–98.

[bb30] Kyriakou, V., Garagounis, I., Vourros, A., Vasileiou, E. & Stoukides, M. (2020). *Joule*, **4**, 142–158.

[bb31] Le Bail, A., Duroy, H. & Fourquet, J. L. (1988). *Mater. Res. Bull.* **23**, 447–452.

[bb32] Lee, P. A., Citrin, P. H., Eisenberger, P. & Kincaid, B. M. (1981). *Rev. Mod. Phys.* **53**, 769–806.

[bb33] Li, Z., Kjølseth, C. & Haugsrud, R. (2015). *J. Membr. Sci.* **476**, 105–111.

[bb34] López-Vergara, A., Porras-Vázquez, J. M., Vøllestad, E., Canales-Vazquez, J., Losilla, E. R. & Marrero-López, D. (2018). *Inorg. Chem.* **57**, 12811–12819. 10.1021/acs.inorgchem.8b02010 30280892

[bb35] Magrasó, A. & Frontera, C. (2016). *Dalton Trans.* **45**, 3791–3797. 10.1039/c5dt04659a 26818222

[bb36] Magrasó, A., Frontera, C., Marrero-López, D. & Núñez, P. (2009). *Dalton Trans.* pp. 10273–10283. 10.1039/b916981b 19921063

[bb37] Magrasó, A. & Haugsrud, R. (2014). *J. Mater. Chem. A*, **2**, 12630–12641.

[bb38] Magrasó, A., Polfus, J. M., Frontera, C., Canales-Vázquez, J., Kalland, L. E., Hervoches, C. H., Erdal, S., Hancke, R., Islam, M. S., Norby, T. & Haugsrud, R. (2012). *J. Mater. Chem.* **22**, 1762–1764.

[bb39] Malerød-Fjeld, H., Clark, D., Yuste-Tirados, I., Zanón, R., Catalán-Martinez, D., Beeaff, D., Morejudo, S. H., Vestre, P. K., Norby, T., Haugsrud, R., Serra, J. M. & Kjølseth, C. (2017). *Nat. Energ.* **2**, 923–931.

[bb40] Marnellos, G. & Stoukides, M. (1998). *Science*, **282**, 98–100. 10.1126/science.282.5386.98 9756486

[bb41] McCarthy, G. J., Fischer, R. D., Johnson, G. G. Jr & Gooden, C. E. (1972). *Solid State Chemistry*, National Bureau of Standards Special Publication No. 364, edited by R. S. Roth & S. J. Schneider Jr, pp. 397–411. Washington, DC: Institute for Materials Research.

[bb42] Morejudo, S. H., Zanón, R., Escolástico, S., Yuste-Tirados, I., Malerød-Fjeld, H., Vestre, P. K., Coors, W. G., Martínez, A., Norby, T., Serra, J. M. & Kjølseth, C. (2016). *Science*, **353**, 563–566. 10.1126/science.aag0274 27493179

[bb43] Ravel, B. & Newville, M. (2005). *J. Synchrotron Rad.* **12**, 537–541. 10.1107/S0909049505012719 15968136

[bb44] Ricote, S., Bonanos, N., Marco de Lucas, M. C. & Caboche, G. (2009). *J. Power Sources*, **193**, 189–193.

[bb45] Ricote, S., Bonanos, N., Wang, H. J. & Haugsrud, R. (2011). *Solid State Ionics*, **185**, 11–17.

[bb46] Rietveld, H. M. (1969). *J. Appl. Cryst.* **2**, 65–71.

[bb47] Ruf, M., Solís, C., Escolástico, S., Dittmeyer, R. & Serra, J. M. (2014). *J. Mater. Chem. A*, **2**, 18539–18546.

[bb48] Schelling, P. K., Phillpot, S. R. & Wolf, D. (2001). *J. Am. Ceram. Soc.* **84**, 1609–1619.

[bb49] Scherb, T. (2011). PhD thesis, Technische Universität Berlin, Germany.

[bb50] Scherb, T., Kimber, S. A. J., Stephan, C., Henry, P. F., Schumacher, G., Escolástico, S., Serra, J. M., Seeger, J., Just, J., Hill, A. H. & Banhart, J. (2016). *J. Appl. Cryst.* **49**, 997–1008.

[bb51] Seeger, J., Ivanova, M. E., Meulenberg, W. A., Sebold, D., Stöver, D., Scherb, T., Schumacher, G., Escolástico, S., Solís, C. & Serra, J. M. (2013). *Inorg. Chem.* **52**, 10375–10386. 10.1021/ic401104m 24000891

[bb52] Shannon, R. D. (1976). *Acta Cryst.* A**32**, 751–767.

[bb53] Teo, B. K. & Lee, P. A. (1979). *J. Am. Chem. Soc.* **101**, 2815–2832.

[bb54] Toby, B. H. (2006). *Powder Diffr.* **21**, 67–70.

[bb55] Toby, B. H. & Von Dreele, R. B. (2013). *J. Appl. Cryst.* **46**, 544–549.

[bb56] Trunov, V. K. (1968). *Russ. J. Inorg. Chem.* **13**, 491–493.

[bb57] Vøllestad, E., Strandbakke, R., Tarach, M., Catalán-Martínez, D., Fontaine, M. L., Beeaff, D., Clark, D. R., Serra, J. M. & Norby, T. (2019). *Nat. Mater.* **18**, 752–759. 10.1038/s41563-019-0388-2 31160804

[bb58] Vøllestad, E., Vigen, C. K., Magrasó, A. & Haugsrud, R. (2014). *J. Membr. Sci.* **461**, 81–88.

[bb59] Willmott, P. R., Meister, D., Leake, S. J., Lange, M., Bergamaschi, A., Böge, M., Calvi, M., Cancellieri, C., Casati, N., Cervellino, A., Chen, Q., David, C., Flechsig, U., Gozzo, F., Henrich, B., Jäggi-Spielmann, S., Jakob, B., Kalichava, I., Karvinen, P., Krempasky, J., Lüdeke, A., Lüscher, R., Maag, S., Quitmann, C., Reinle-Schmitt, M. L., Schmidt, T., Schmitt, B., Streun, A., Vartiainen, I., Vitins, M., Wang, X. & Wullschleger, R. (2013). *J. Synchrotron Rad.* **20**, 667–682. 10.1107/S0909049513018475 PMC3747948 23955029

[bb60] Yamazoe, S., Hitomi, Y., Shishido, T. & Tanaka, T. (2008). *J. Phys. Chem. C*, **112**, 6869–6879.

